# Correlations of PTEN and ERG Immunoexpression in Prostate Carcinoma and Lesions Related to Its Natural History: Clinical Perspectives

**DOI:** 10.3390/cimb45040181

**Published:** 2023-03-25

**Authors:** Olga Voulgari, Dimitrios Goutas, Alexandros Pergaris, Konstantinos Belogiannis, Eirini Thymara, Nikolaos Kavantzas, Andreas C. Lazaris

**Affiliations:** First Department of Pathology, School of Medicine, The National and Kapodistrian University of Athens, “Laikon” General Hospital of Athens, 11527 Athens, Greece

**Keywords:** prostate carcinoma, PTEN, ERG, AMACR, high-grade prostatic intraepithelial neoplasia, intraductal carcinoma of the prostate

## Abstract

**Purpose**: The aim of our study was to observe the associations between the ETS-related gene (*ERG)* and the phosphatase and tensin homolog gene (*PTEN*) immunoexpression in prostate cancer and related lesions and highlight the clinical significance of these findings. **Methods**: We evaluated the immunohistochemical expression of *ERG* and *PTEN* in a series of 151 invasive prostate adenocarcinomas, including low-grade (Gleason grade pattern 3) and high-grade (Gleason grade patterns 4, 5) morphological patterns which corresponded to 45.5% and 54.4% of the cases, respectively. Additionally, we evaluated the immunoexpression of the two markers both in foci of high-grade prostatic intraepithelial neoplasia (HGPIN), as a precursor lesion of cancer, and in foci of intraductal carcinoma of the prostate (IDCP). Finally, to ensure the malignant nature of the prostate glands examined, we employed p63 and alpha-methylacyl-CoA racemase (*AMACR)* expression. **Results**: We found that *PTEN* loss was observed in 50.7%, and *ERG* positivity was detected in 41.8% of our cancerous samples. In HGPIN, *PTEN* loss appeared to be linked with a high-grade adjacent invasive carcinoma component which also displayed *PTEN* loss. As far as IDCP is concerned, *ERG* immunonegativity was correlated with adjacent high-grade invasive cancer, which was also *ERG* immunonegative. **Conclusions**: Our findings suggest that the clonal expansion of invasive cancer appears to be associated with distinct immunophenotypic cellular alterations of both early and late cancer-related histological lesions. Patients with *PTEN* loss in HGPIN in prostate biopsies should be closely monitored due to the increased likelihood of having an associated invasive high-grade carcinoma that may have not been sampled. Given the clinical significance that derives from *PTEN* expression in HGPIN lesions, we suggest the routine use of *PTEN* immunohistochemistry in prostate cancer biopsies in which HGPIN is the only finding.

## 1. Introduction

Prostate cancer represents the most common malignancy among men in the western world [1] and a highly biologically variable disease. In the USA, 268,490 new cases of prostate cancer were recorded during 2022, comprising 14% of all new cancer cases, and it is estimated that approximately 12.6 percent of men will be diagnosed with prostate cancer at some point during their lifetime [1]. Gleason scoring [1,2] of prostate cancer tissues remains the most widely applied and robust utility for assessing tumor progression and aggressiveness, as well as a trustworthy system upon which most decisions regarding patients’ management are carried out. A considerable proportion of prostate cancers display a relatively indolent course without significant associated morbidity, as 5-year survival rates amount to 96.8%, according to the National Cancer Institute [1]. However, some cases follow a highly aggressive clinical course, leading to polymetastatic disease which eventually becomes treatment-resistant and ultimately lethal. Taking into account the considerable prevalence of prostate cancer in the male population as well, it becomes evident that, besides the Gleason grading pattern [2,3], there is an urgent need for the identification of novel predictive biomarkers to differentiate low-risk patients from those at increased risk for disease progression and metastasis. Despite the fact that our knowledge of genomic markers has greatly improved over the years, there is still a universal lack of predictive biomarkers that could be reliably used in clinical practice [4]. 

The *ERG* is located at chromosome 21q22.2 and it is a member of the erythroblast transformation-specific (ETS) family of transcriptions factors, a group of highly conserved molecules with an important role in multiple cellular events, including embryonic development, proliferation, and differentiation [2]. 

Under normal circumstances, *ERG* is not expressed in the epithelial cells of the prostate. However, increased expression is frequently present in patients with prostate cancer [3,4]. The over-expression of *ERG* is highly attributed to the *TMPRSS2: ERG* fusion, a recurrent chromosomal rearrangement that represents the most common molecular alteration of prostate cancer, approximately occurring in one-half of the cases [2,5]. *TMRSS2: ERG* fusion can occur due to genomic translocations or due to interstitial deletions of the intergenic region between the two genes. This genomic event results in the fusion of the *ERG* proto-oncogene with the androgen-driven promoter of the *TMPRSS2*, setting the transcription of the former under hormonal affection and resulting in its over-expression in cancer cells [5]. The exact molecular mechanisms underlying the pathogenic role of *TMPRSS2: ERG* fusion in prostate cancer are yet to be fully uncovered, but there is evidence suggesting its association with the clinical course of the disease [3,6]. 

The *PTEN* gene is located in chromosome 10, encoding an enzymatically active molecule that acts as a phosphatase, which impedes the activity of the PI3K/Akt pathway. *PTEN* has been shown to constitute the most frequently inactivated tumor suppressor gene in primary prostate cancer, and its loss has been associated with disease progression in both hormone-naive and castration-resistant prostate cancer [7]. Various methods, including immunohistochemistry, in situ and array comparative genomic hybridization, have been used to highlight the loss of *PTEN* function in prostate cancer [7,8]. Genomic deletion is the most commonly found mechanism of *PTEN* alteration, whereas point mutations are less frequent. A loss of *PTEN* expression in pathology specimens, as evidenced by the use of immunohistochemistry staining, has been correlated with low *PTEN* signaling at the molecular level, regarding DNA and mRNA [9,10]. Immunohistochemistry-targeting *PTEN* protein stains the nucleus and the cytoplasm of the basal and luminal prostatic cells, and loss of this staining pattern is frequently found in prostate cancer. Higher rates of *PTEN* loss have been associated with disease severity and progression along with poorer clinical outcomes [11].

The fusion of the androgen-regulated serine protease *TMPRSS2* with the ETS family transcription factor *ERG* along with the inactivating rearrangements of the tumor suppressor gene phosphatase and tensin homolog (*PTEN*) are among the most common genetic alterations in prostate cancer [5]. *PTEN* loss has been linked to poor overall survival, adverse pathological findings and the development of castration-resistant and metastatic disease [6,7,8]. Meanwhile, *TMPRSS2-ERG* fusions represent an early event in prostate carcinogenesis and are present in approximately 36–78% of cases [9,10,11]. Although *PTEN* loss is less prevalent, it likely represents a more clinically relevant genetic rearrangement than *TMPRSS2-ERG* fusion in prostate cancer. The detection of the aforementioned alterations through immunohistochemistry has been proven as a reliable and useful technique in detecting the expression of *ERG*, a surrogate marker for *TMPRSS2-ERG* fusion, and of *PTEN* [9,12,13,14,15].

Whereas the overexpression of *ERG* alone does not constitute a determinant for disease progression or overall survival [14,15], it could indicate the initiating trigger for the gene fusion upon other cancer cell regulatory pathways, such as a loss of *PTEN*, and thus reflect disease aggressiveness [16,17,18,19,20,21]. On the other hand, the loss of *PTEN* alone seems to add some value in predicting patient outcomes [22,23]. Nevertheless, there is more clinical significance when considering the combined effect of *TMPRSS2-ERG* fusion and *PTEN* deletion than either one alone [24,25]. Studies have shown that the *ERG* fusion-positive–*PTEN*-negative prostate cancers display unfavorable disease outcomes such as earlier biochemical recurrence [16,26] and cancer progression [27,28]. However, to date, there are few studies depicting disease-specific survival based on *ERG* and *PTEN* status.

In the present study, we aimed to observe the patterns of *ERG* immunoexpression and *PTEN* immunohistochemical loss in prostate cancer precursor lesions (HGPIN), invasive prostate carcinoma, as well as in IDCP in correlation with the immunohistochemical expression in the adjacent invasive carcinoma.

## 2. Materials and Methods

### 2.1. Patient Material Collection and Characterization

Following systematic research of the database of our department, formalin-fixed paraffin-embedded (FFPE) tissues from 151 radical prostatectomy specimens of Gleason scores 6–10 were retrieved from the pathology laboratory archives of the First Department of Pathology, School of Medicine, NKUA, “Laikon” General Hospital in Athens, Greece. Out of the 151 cases, 17 were excluded due to technical problems. Low-grade (Gleason grade pattern 3) prostate cancers represented 45.7% of our cases, whereas high-grade (Gleason grade patterns 4, 5) prostate cancers were 54.3% of the specimens (Table 1). HGPIN was detectable in 113 cases and IDCP in 35 cases. All tissue samples selected regarded retrospective cases of our department. Inclusion criteria were the presence of prostate acinar adenocarcinoma, HGPIN and IDCP.

### 2.2. Immunohistochemistry Procedure and Evaluation

Immunohistochemistry was carried out using standard procedures in all tested specimens. Firstly, the sections were stained with the following antibodies on a Dako system, according to the manufacturer’s protocol. The sections were stained based on the double-staining protocol of Leica Biosystems in the Bond-III fully automated stainer, with antibodies against p63 (clone 4A4 Biocare/at dilution 1:100, Pacheco, CA 94553, USA), *AMACR* (monoclonal rabbit, clone 13H4, Dako/at dilution 1:50, Santa Clara, CA 95051, USA), *ERG* (clone EP111, ready to use, Dako, Santa Clara, CA 95051, USA) and *PTEN* (monoclonal mouse, clone 6H2.1, Dako/at dilution 1:200, Santa Clara, CA 95051, USA). Antigen retrieval was performed at pH 6. The Envision (Dako) visualization system was used. DAB (3,3-diaminobenzidine) was used as a chromogen and hematoxylin as counterstain. A double staining protocol was used both for *PTEN* (red chromogen)-*ERG* (brown chromogen) and for *AMACR* (red chromogen)-*p63* (brown chromogen). The evaluation of the slides was carried out by two experienced pathologists (D.G. and A.C.L) independently who were initially blinded to the clinicopathological data of the patients as well as to each other’s results. After a literature review [29], a tissue specimen was considered to have *PTEN* protein loss if the intensity of cytoplasmic and nuclear staining was noticeably reduced or completely negative across >10% of tumor cells in relation to the surrounding benign glands or stroma, which were used as internal positive controls. In cases where *PTEN* was lost in >10 and <100% of the tumor cells, the specimen was considered to have heterogeneous *PTEN* loss (score 1). Otherwise, when the specimen showed *PTEN* loss in 100% of the tumor glands, it was considered to have homogeneous *PTEN* loss (score 0). *PTEN* preservation across the entirety of the specimen was considered as score 2. *ERG* expression was considered to be positive when any tumor cells displayed nuclear *ERG* expression and negative if there was a complete absence of *ERG* expression. Endothelial cells were used as internal positive controls [29]. Each immunomarker was separately assessed in each Gleason pattern. Double staining with p63 (brown chromogen) and *AMACR* (red chromogen) was performed to confirm the diagnosis of prostate adenocarcinoma. *AMACR* was considered positive in the carcinoma when it displayed a strong granular cytoplasmic pattern in the luminal cells of the neoplastic prostate glands, whereas the absence of p63 in the same glands was considered diagnostic. The nuclear staining of the basal cell layer of the adjacent normal prostate glands was used as an internal control. Our group then proceeded to correlate the staining patterns of the aforementioned markers with a multitude of histopathological characteristics, including the Gleason score of prostate carcinoma specimens and the presence of HGPIN and IDCP. As HGPIN we define the proliferation of atypical secretory cells within prostatic glands. It is thought that HGPIN is the earliest histologically recognizable precursor of invasive adenocarcinoma of the prostate. On the other hand, IDCP is a neoplastic epithelial proliferation involving pre-existing, generally expanded, duct-acinar structures and characterized by architectural and cytological atypia beyond what is acceptable for HGPIN^2^.

### 2.3. Statistical Analysis

Statistical analysis was performed with IBM-SPSS v26. The level of statistical significance was set at 5% (α = 0.05). Pearson’s χ^2^ test (Pearson’s chi-square test for categorical data) with continuity correction for 2 × 2 tables was performed in order to check for a statistically significant relation between studied markers.

## 3. Results

*AMACR* was totally negative in 4.5% (6/134) of cancer samples and positive in the remaining 95.5% (128/134). *PTEN* displayed homogeneous loss (score 0) in 50.7% (68/132) of our cases, heterogeneous loss (score 1) in 41.8% (56/132) of the cases and expression preservation (score 3) in the remaining 7.5% (10/134). Finally, *ERG* was negative in 58.2% (78/134) of our cases and positive in 41.8% (56/134) of our cases (Table 2). No other associations of *ERG* and *PTEN* expression with clinicopathological parameters provided statistically significant results.

### 3.1. ERG and PTEN Associations in Prostate Carcinoma

**Figure 1:**
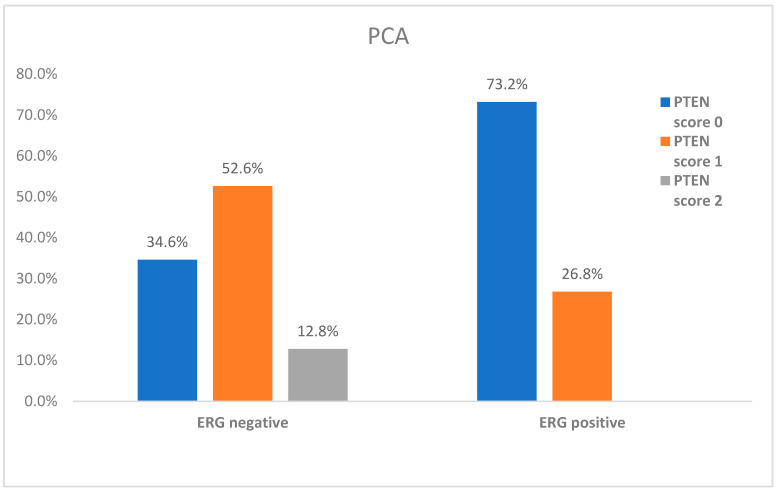
Interrelations between *ERG* and *PTEN* immunoexpression in prostate cancer.

**Figure 2:**
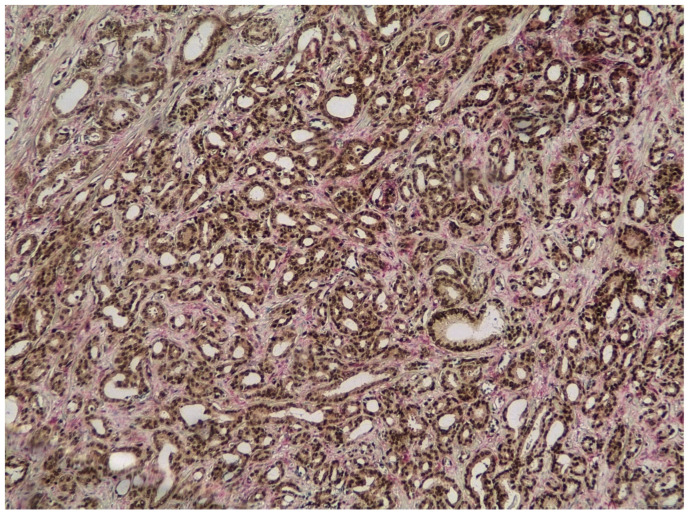
*ERG* nuclear immunopositivity combined with complete *PTEN* loss in cancerous tissue (×100).

Statistical analysis of *PTEN* and *ERG* expression in prostate carcinoma provided statistically significant results (*p* < 0.0001) (Figure 1). More specifically, when *ERG* was negative in prostate carcinoma, *PTEN* displayed a heterogeneous loss (score 1). Meanwhile, the positive expression of *ERG* proved to be significantly associated with a homogeneous loss (score 0) of *PTEN* in prostate cancer (Figure 2).

### 3.2. Association of ERG Expression with Gleason Grade Pattern in Prostate Cancer

**Figure 3:**
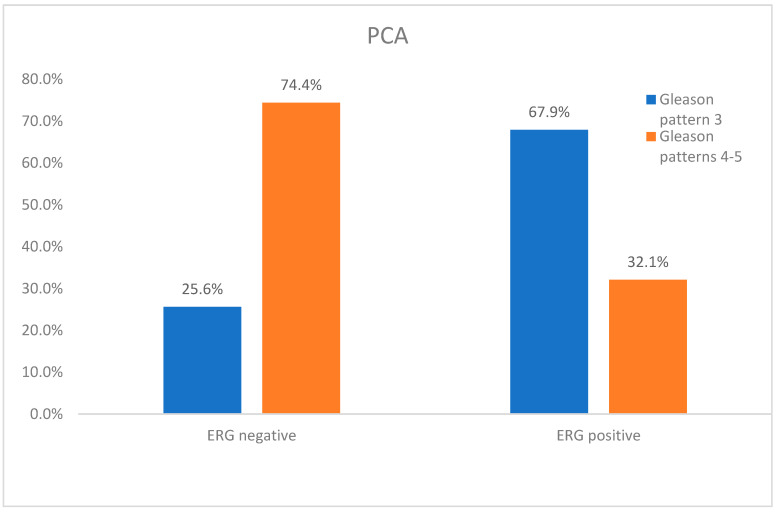
*ERG* immunoexpression in relation to Gleason grade pattern of prostate cancer.

**Figure 4:**
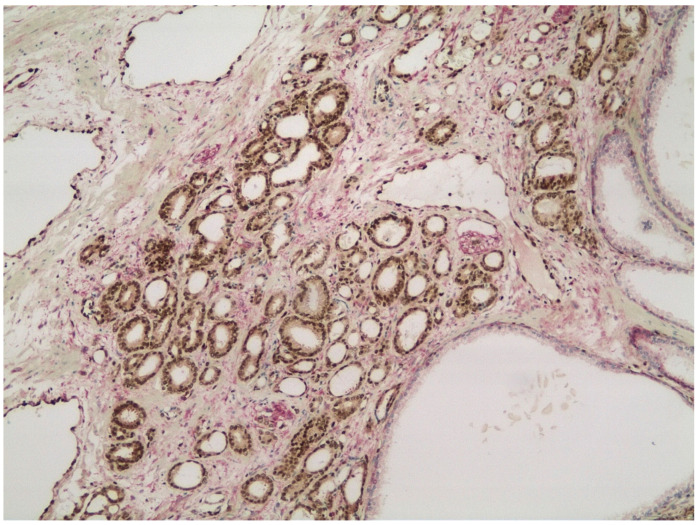
*ERG* nuclear immunopositivity in prostate acinar adenocarcinoma of Gleason grade pattern 3 (×100).

The Gleason grade pattern displayed a statistically significant association with the expression of *ERG* (*p* < 0.0001) (Figure 3). Characteristically, high-grade carcinomas of Gleason grade patterns 4 and 5 were ERG-negative, whereas low-grade prostate carcinomas of Gleason grade pattern 3 were *ERG-positive* (Figure 4).

### 3.3. PTEN Expression in HGPIN and Its Association with the Adjacent Invasive Prostate Cancer

**Figure 5:**
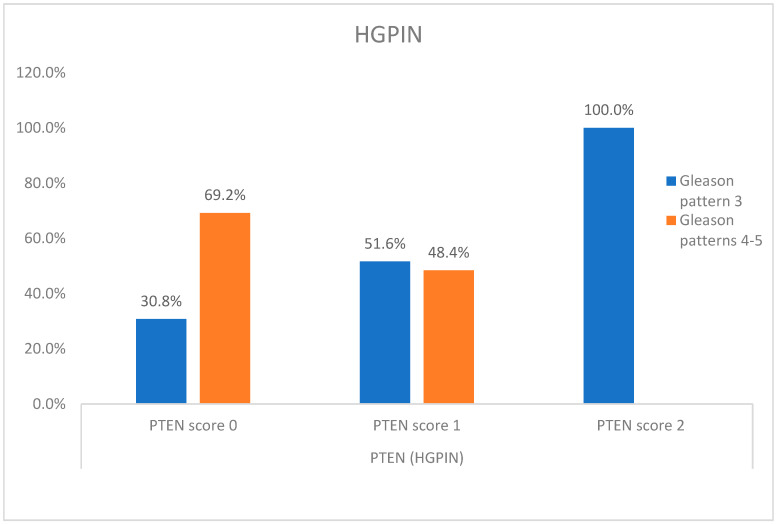
*PTEN* scores in HGPIN in relation to *PTEN* score and Gleason grade pattern of the adjacent carcinoma.

**Figure 6:**
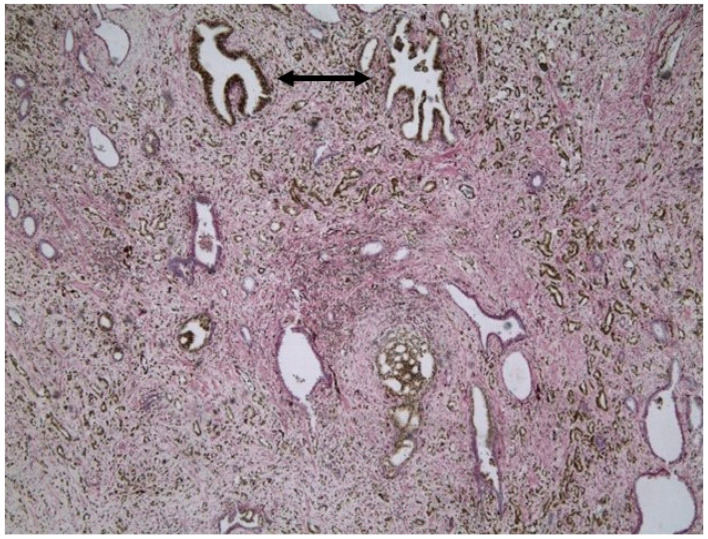
Two HGPIN foci with total *PTEN* loss and simultaneous *ERG* positivity (arrows) and adjacent high-grade invasive carcinoma [in the form of the cribriform cancerous gland (pattern 4) and single cells (pattern 5)] with total *PTEN* loss (×40).

**Figure 7:**
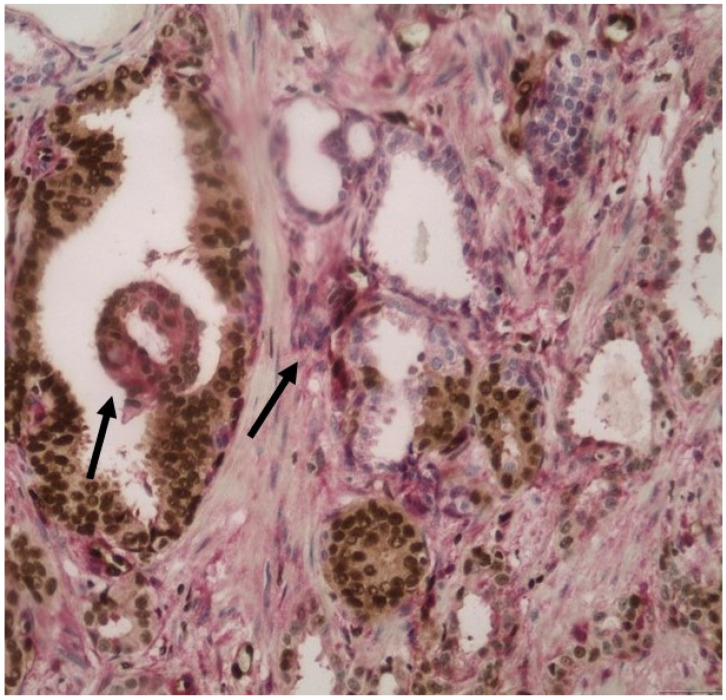
*PTEN* positivity in HGPIN (left side of the image) with concomitant *PTEN* positivity in adjacent low-grade invasive carcinoma (arrows showing *PTEN* positive focus of HGPIN and invasive glands). Simultaneous *ERG* nuclear expression in both HGPIN and invasive cancer (×200).

In cases with HGPIN, the Gleason grade pattern of the co-existent adjacent prostate carcinoma demonstrated statistically significant differences when compared to *PTEN* expression in the HGPIN foci (*p* = 0.05) (Figure 5). Cases showing *PTEN* homogeneous loss in HGPIN (score 0) were associated with high-grade adjacent invasive carcinoma (Gleason grade pattern 4 or 5) and a *PTEN* score of 0 (Figure 6). In cases where *PTEN* was preserved in the HGPIN (score 2), the adjacent prostate carcinoma had low-grade features (Gleason grade pattern 3) and retained its *PTEN* expression (Figure 7). When the *PTEN* was heterogeneously lost in HGPIN (score 1), then 51% of adjacent invasive prostate cancer was low-grade and had a *PTEN* score of 2.

### 3.4. ERG Expression in IDCP and Its Association with the Adjacent Invasive Prostate Carcinoma

**Figure 8:**
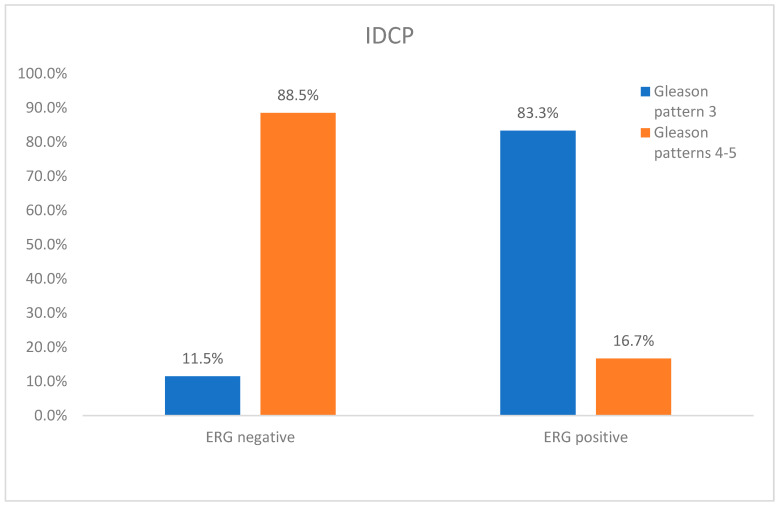
*ERG* immunoexpression of IDCP in relation to *ERG* expression and Gleason grade pattern of the adjacent invasive carcinoma.

**Figure 9:**
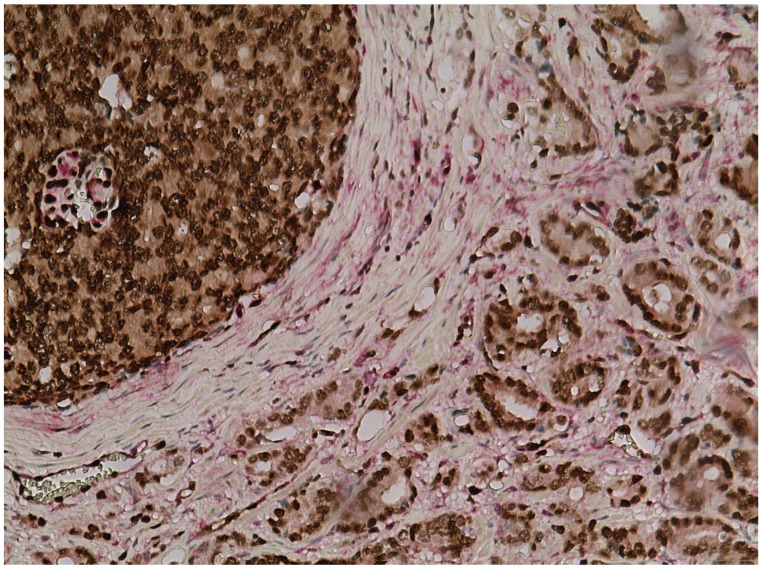
Simultaneous *ERG* nuclear immunopositivity in intraductal carcinoma and in adjacent low-grade invasive carcinoma (×200).

In IDCP, the Gleason grade pattern of the adjacent invasive component showed statistically significant differences when analyzed in relation to the *ERG* expression (*p* = 0.002) (Figure 8). More specifically, when IDCP expressed ERG, the adjacent invasive prostate cancer was also ERG-positive with low-grade features (Figure 9). On the contrary, when IDCP was *ERG*-negative, the adjacent prostate cancer was also *ERG*-negative and displayed high-grade morphology. As such, we observed that the pattern of *ERG* staining in IDCP is identical to that of the adjacent invasive cancer.

### 3.5. AMACR Expression in Prostate Cancer and Its Association with PTEN

*AMACR* expression in prostate cancer was correlated with *PTEN* expression in prostate cancer (*p* = 0.039). More precisely, when *AMACR* was negative, the *PTEN* displayed a heterogeneous loss (score 1) expression pattern, whereas when *AMACR* was positive, *PTEN* demonstrated a homogeneous loss (score 0).

## 4. Discussion

*PTEN* deletion and *ERG* rearrangement exhibit a significant role in the pathogenesis and clinical course of prostate cancer, and both represent frequent genetic alterations [8,28,30,31,32]. Among others, the contribution of *PTEN* deletion to prostate cancer tumorigenesis and progression was underlined in a large study that incorporated more than 4.5 thousand specimens and correlated its presence to advanced tumor stages, higher Gleason score grades, positive lymph nodes and androgen-independent disease, linking it to an overall aggressive tumor phenotype [12]. The synergistic action of *ERG* rearrangement in carcinogenesis has also been explored, with researchers demonstrating the frequency of coexistence of the two phenomena in malignant tissues. Specifically, TMPRSS2-ERG fusions, which constitute by far the most frequent aberration (>97%), were shown to strongly associate with *PTEN* deletions [7,13,14].

In the current study, we demonstrated that homogeneous *PTEN* loss was associated with immunohistochemical *ERG* positivity; the latter being mostly linked to low-grade prostate carcinomas. On the contrary, *PTEN* heterogeneous loss was statistically linked with *ERG* negativity, which was in turn related to high-grade prostate carcinomas.

Genomic *PTEN* loss has been associated, as previously mentioned, with prostate cancer progression, aggressiveness and poor prognosis [33,34,35]. Among others, researchers have linked this molecular feature with increased tumor grade and even increased lethality in patients who underwent radical prostatectomy [33,34], with the risk being more pronounced when tumors exhibited a concurrent loss of *ERG* immunoexpression [35]. In our study, we observed that *PTEN* homogeneous loss was observed in 50.7% of cases which has been reported to correspond to *PTEN* gene homozygous deletion [18]. Meanwhile, *PTEN* heterogeneous loss was present in 41.8% of cancer specimens, which most likely corresponds to a heterogeneous or subclonal *PTEN* gene deletion [18]. Nevertheless, we did not manage to establish an association of *PTEN* loss (when evaluating *PTEN* loss alone) with prostate carcinoma aggressiveness, perhaps due to the relatively small number of our specimens. On the contrary, when assessing the *ERG–PTEN* association, we observed that *PTEN* homogeneous loss was linked to *ERG* positivity which was mostly found in low-grade prostate carcinomas, and *PTEN* heterogeneous loss was linked to a negative *ERG* expression which constitutes a key finding in high-grade carcinomas. Therefore, we can support that immunohistochemical homogeneous *PTEN* loss is predominantly linked to *ERG* fusions (*ERG*-positive immunostaining), a mechanism mostly seen in prostate adenocarcinomas of low grade. It has been claimed that *PTEN* alterations typically develop subsequent to *ERG* fusions [10]. More specifically, Krohn et al. in the aforementioned study performed a tissue microarray analysis for *PTEN* alterations (including deletions and breaks) and compared *PTEN* and *ERG* status in the same tumor areas [10]. The data from their study showed the substantial heterogeneity in *PTEN* aberrations in prostate cancer and advocates that *ERG* activation is a key driver of such abnormalities. On the contrary, *PTEN* alterations, as a primary lesion in cancer, did not display an increased risk for developing *TMPRSS2: ERG* fusions [10].

PTEN and *ERG* immunoexpression has been investigated with the aim to explore the utility of the aforementioned biomarkers in the challenging task of distinguishing between HGPIN and IDCP in the setting of prostate biopsies. In our study, a *PTEN* immunostaining pattern in HGPIN was identical to that of the adjacent invasive carcinoma tissues when the *PTEN* pattern was either score 0 or 2 in high-grade or low-grade carcinomas, respectively. Therefore, we demonstrated, for the first time to our knowledge, that HGPIN cases showing homogeneous *PTEN* loss (score 0) are associated with the presence of an adjacent high-grade invasive carcinoma focus, whereas the preservation of *PTEN* in the HGPIN component was linked to an adjacent low-grade invasive prostate carcinoma. This is partly explained as HGPIN represents a precursor lesion, so it is speculated that in these cases, the same neoplastic clone gives rise to and progresses to invasive prostate carcinoma with similar genomic characteristics. The same applies for the cases of *PTEN* preservation in the HGPIN. However, what is of particular interest is the fact that 51% of HGPIN cases that displayed heterogeneous *PTEN* loss were linked to an adjacent low-grade invasive prostate adenocarcinoma. In these HGPIN cases with heterogeneous *PTEN* loss, the adjacent carcinoma displayed *PTEN* expression preservation (score 2) only in its low-grade form. So, complementary to what we observed in the former cases, in these *PTEN* score 1 HGPIN cases, we see that probably the HGPIN clone that gives rise to the adjacent low-grade invasive carcinoma is the one that preserves *PTEN* expression. These findings further underpin the model of clonal progression in prostate cancer [36]. With regard to the clone of low-grade adjacent cancer, cancerous cells are likely to arise from those cells that preserve *PTEN* immunoexpression within HGPIN; on the contrary, high-grade cancer cells might derive from those cells of HGPIN which lack *PTEN* immunoexpression. The individual cells within a precursor lesion acquire potentially additional genomic abnormalities, resulting in subclonal tumor cell populations and invasion [36].

As far as *ERG* expression is concerned, we also observed an association with IDCP, a late event in prostate cancer progression. The incidence of IDCP amounts to an estimated 20% of radical prostatectomies [15]. The presence of IDCP constitutes a very important finding in prostate carcinoma, as it heavily influences patients’ prognosis and management. A multitude of studies has underlined its association with poor outcomes [15,16,17,18] as well as with many adverse clinicopathological characteristics, including positive lymph nodes [19,20], distant metastasis [16,21] and castration-resistant disease [22]. Researchers have attempted to shed light on the link between *PTEN* loss and *ERG* rearrangements with IDPC, as all three phenomena represent important factors in the progression of prostate adenocarcinoma. More specifically, two studies reported the coexistence of IDPC with *ERG* rearrangements in 94% [23] and 75% [24] of cases, while *PTEN* loss has been observed in 84% [25] and 72% [26] of IDCP specimens. Characteristically, in our study, when IDCP expressed *ERG*, the adjacent invasive carcinoma was also *ERG*-positive and paradoxically low-grade, whereas when IDCP lacked *ERG* expression, the abutting invasive component was *ERG*-negative and high-grade. According to the most widely accepted IDCP theory, IDCP is by definition a high-grade lesion which represents an end-stage spread of high-grade invasive carcinoma into ducts and acini [2,37]. Therefore, it is reasonable that the ducts colonized by an elsewhere-developed invasive component will show the same clonal neoplastic traits. According to our findings, the *ERG* immunopositivity of the IDCP-adjacent low-grade invasive carcinoma cannot be a fundamental immunophenotypic characteristic related to the cancerous clone within IDCP.

Moreover, although we were not able to obtain a statistically significant result, probably due to the small number of our cohort, we observed that 43.8% of our IDCP cases displayed a homogeneous *PTEN* loss. This is in line with findings by Haffner et al., according to which, IDCP occurs subsequent to invasive carcinoma development [36].

Finally, *AMACR* was positive in 95.5% of our prostate cancer samples and negative in the remaining 4.5%. Interestingly, *AMACR*-negative expression in prostate cancer was associated with a heterogeneous *PTEN* loss, while its positive expression was linked to *PTEN* homogeneous loss. There have been a few studies discussing the potential oncogenic role of *AMACR* in prostate adenocarcinomas [38,39], so in conjunction with its strong association with *PTEN* homogeneous loss, it could prove to be an important candidate biomarker for prostate cancer progression and prognosis apart from its well-established diagnostic significance. This is in keeping with other groups of researchers that have also linked increased AMACR expression to higher-grade tumors [27,28] as well as to patients’ age [27]. Moreover, AMACR-positive HGPIN lesions in biopsies that did not harbor prostate carcinoma have exhibited a five-fold increased risk of cancer diagnosis in subsequent biopsies, compared to patients with AMACR-negative HGPIN [29]. It is evident, however, that further studies are needed in order to establish *AMACR* as a strong predictor of clinical outcome.

All in all, our study confirmed the strong relationship between *ERG* rearrangement and *PTEN* deletion reinforcing the theory of an interactive or cooperative role of the two phenomena in the biology of prostate cancer. Furthermore, we displayed a possible clonal relationship between the neoplastic cells of precursor lesions (HGPIN) and the adjacent invasive carcinoma based on *PTEN* expression patterns, and among IDCP and the abutting invasive cancer, based on *ERG* expression. According to our findings and the existing knowledge regarding the biological role of *PTEN* loss and *ERG* rearrangements, we suggest that patients with HGPIN in prostate biopsies displaying homogeneous *PTEN* loss would benefit from a more intensive clinical follow-up. In those patients, should a co-existent invasive cancer be found, it would most likely be of high grade and thus clinically significant. Our findings underpin our understanding of the sequence of molecular and morphological events and of complex clonal relationships that arise during prostate cancer progression. Moreover, we provide evidence that would help optimize the histological risk stratification of prostate cancer patients based on the immunohistochemical evaluation of precursor lesions in prostate biopsies. Among the limitations of our study is the lack of a prolonged follow-up of more than 5 years, since the 5-year survival rate of prostate cancer is nearly 100% [40]. Furthermore, the fact that we did not use in situ molecular techniques (hybridization) in order to establish our immunohistochemical results represents another limitation of our study.

## 5. Conclusions

Overall, it is evident that disorders of *PTEN* gene expression seem to be associated with an early stage of prostate carcinoma progression as their significance appears to be focused on HGPIN lesions of our samples. On the contrary, *ERG* immunoexpression appears to correlate with the tumor grade of invasive carcinoma with its positivity linked to low-grade tumors. What appears, however, to be of importance and clinical significance derives from *PTEN* expression in HGPIN lesions. Therefore, we suggest the routine use of *PTEN* immunohistochemistry in prostate cancer biopsies when HGPIN lesions are encountered as the only finding.

## Figures and Tables

**Table 1:** Descriptive statistics regarding Gleason grade pattern in our prostate cancer cases.

		Number of Cases	%
**Grade**	**3**	61	45.5%
	**4, 5**	73	54.5%
	**Total**	134	100.0%

**Table 2:** Frequencies of study variables in our study with regard to prostate carcinoma.

Prostate Carcinoma		Number of Cases	%
**P63**	**Negative**	134	100%
**AMACR**	**Negative**	6	4.5%
	**Positive**	128	95.5%
**PTEN (Pca)**	**Score 0**	68	50.7%
	**Score 1**	56	41.8%
	**Score 2**	10	7.5%
**ERG (Pca)**	**Negative**	78	58.2%
	**Positive**	56	41.8%

## Data Availability

No new data were generated.

## References

[1] Center M.M., Jemal A., Lortet-Tieulent J., Ward E., Ferlay J., Brawley O., Bray F. (2012). International Variation in Prostate Cancer Incidence and Mortality Rates. Eur. Urol..

[2] Destouni M., Lazaris A.C., Tzelepi V. (2022). Cribriform Patterned Lesions in the Prostate Gland with Emphasis on Differential Diagnosis and Clinical Significance. Cancers.

[3] van Leenders G.J., van der Kwast T.H., Grignon D.J., Evans A.J., Kristiansen G., Kweldam C.F., Litjens G., McKenney J.K., Melamed J., Mottet N. (2020). The 2019 International Society of Urological Pathology (ISUP) Consensus Conference on Grading of Prostatic Carcinoma. Am. J. Surg. Pathol..

[4] Boström P.J., Bjartell A.S., Catto J.W., Eggener S.E., Lilja H., Loeb S., Schalken J., Schlomm T., Cooperberg M.R. (2015). Genomic Predictors of Outcome in Prostate Cancer. Eur. Urol..

[5] Zhao L., Yu N., Guo T., Hou Y., Zeng Z., Yang X., Hu P., Tang X., Wang J., Liu M. (2014). Tissue Biomarkers for Prognosis of Prostate Cancer: A Systematic Review and Meta-analysis. Cancer Epidemiol. Biomark. Prev..

[6] Mithal P., Allott E., Gerber L., Reid J., Welbourn W., Tikishvili E., Park J., Younus A., Sangale Z., Lanchbury J.S. (2014). PTEN loss in biopsy tissue predicts poor clinical outcomes in prostate cancer. Int. J. Urol..

[7] Fontugne J., Lee D., Cantaloni C., Barbieri C., Caffo O., Hanspeter E., Mazzoleni G., Palma P.D., Rubin M., Fellin G. (2014). MP24-13 Withdrawn: Recurrent Prostate Cancer Genomic Alterations Predict Response to Brachytherapy Treatment. J. Urol..

[8] Krohn A., Diedler T., Burkhardt L., Mayer P.-S., De Silva C., Meyer-Kornblum M., Kötschau D., Tennstedt P., Huang J., Gerhäuser C. (2012). Genomic Deletion of PTEN Is Associated with Tumor Progression and Early PSA Recurrence in ERG Fusion-Positive and Fusion-Negative Prostate Cancer. Am. J. Pathol..

[9] Shah R.B., Bentley J., Jeffery Z., DeMarzo A.M. (2015). Heterogeneity of PTEN and ERG expression in prostate cancer on core needle biopsies: Implications for cancer risk stratification and biomarker sampling. Hum. Pathol..

[10] Krohn A., Freudenthaler F., Harasimowicz S., Kluth M., Fuchs S., Burkhardt L., Stahl P., Tsourlakis M.C., Bauer M., Tennstedt P. (2014). Heterogeneity and chronology of PTEN deletion and ERG fusion in prostate cancer. Mod. Pathol..

[11] Tomlins S.A., Rhodes D.R., Perner S. (2005). Recurrent fusion of TMPRSS2 and ETS transcription factor genes in prostate cancer. Science.

[12] Chaux A., Peskoe S.B., Gonzalez-Roibon N., Schultz L., Albadine R., Hicks J., De Marzo A.M., A Platz E., Netto G.J. (2012). Loss of PTEN expression is associated with increased risk of recurrence after prostatectomy for clinically localized prostate cancer. Mod. Pathol..

[13] Lotan T.L., Gurel B., Sutcliffe S., Esopi D., Liu W., Xu J., Hicks J.L., Park B.H., Humphreys E., Partin A.W. (2011). PTEN Protein Loss by Immunostaining: Analytic Validation and Prognostic Indicator for a High Risk Surgical Cohort of Prostate Cancer Patients. Clin. Cancer Res..

[14] Pettersson A., Graff R.E., Bauer S.R. (2012). The TMPRSS2:ERG rearrangement, ERG expression, and prostate cancer outcomes: A cohort study and meta-analysis. Cancer Epidemiol. Biomark. Prev..

[15] Minner S., Enodien M., Sirma H., Luebke A.M., Krohn A., Mayer P.S., Simon R., Tennstedt P., Müller J., Scholz L. (2011). ERG Status Is Unrelated to PSA Recurrence in Radically Operated Prostate Cancer in the Absence of Antihormonal Therapy. Clin. Cancer Res..

[16] Yoshimoto M., Joshua A.M., Cunha I.W., A Coudry R., Fonseca F.P., Ludkovski O., Zielenska M., A Soares F., A Squire J. (2008). Absence of TMPRSS2:ERG fusions and PTEN losses in prostate cancer is associated with a favorable outcome. Mod. Pathol..

[17] Leinonen K.A., Saramäki O.R., Furusato B., Kimura T., Takahashi H., Egawa S., Suzuki H., Keiger K., Hahm S.H., Isaacs W.B. (2013). Loss of PTEN Is Associated with Aggressive Behavior in ERG-Positive Prostate Cancer. Cancer Epidemiol. Biomark. Prev..

[18] Gumuskaya B., Gurel B., Fedor H.L., Tan H.-L., Weier C.A., Hicks J.L., Haffner M.C., Lotan T., De Marzo A.M. (2013). Assessing the order of critical alterations in prostate cancer development and progression by IHC: Further evidence that PTEN loss occurs subsequent to ERG gene fusion. Prostate Cancer Prostatic Dis..

[19] Carver B.S., Tran J., Gopalan A., Chen Z., Shaikh S., Carracedo A., Alimonti A., Nardella C., Varmeh S., Scardino P.T. (2009). Aberrant ERG expression cooperates with loss of PTEN to promote cancer progression in the prostate. Nat. Genet..

[20] Hoogland A.M., Jenster G., van Weerden W.M., Trapman J., van der Kwast T., Roobol M.J., Schröder F.H., Wildhagen M.F., van Leenders G.J. (2012). ERG immunohistochemistry is not predictive for PSA recurrence, local recurrence or overall survival after radical prostatectomy for prostate cancer. Mod. Pathol..

[21] Xu B., Chevarie-Davis M., Chevalier S., Scarlata E., Zeizafoun N., Dragomir A., Tanguay S., Kassouf W., Aprikian A., Brimo F. (2014). The prognostic role of ERG immunopositivity in prostatic acinar adenocarcinoma: A study including 454 cases and review of the literature. Hum. Pathol..

[22] Cuzick J., Yang Z.H., Fisher G., Tikishvili E., Stone S., Lanchbury J.S., Camacho N., Merson S., Brewer D., on behalf of the Transatlantic Prostate Group (2013). Prognostic value of PTEN loss in men with conservatively managed localised prostate cancer. Br. J. Cancer.

[23] Barnett C.M., Heinrich M.C., Lim J., Nelson D., Beadling C., Warrick A., Neff T., Higano C.S., Garzotto M., Qian D. (2014). Genetic Profiling to Determine Risk of Relapse-Free Survival in High-Risk Localized Prostate Cancer. Clin. Cancer Res..

[24] Reid A.H.M., Attard G., Ambroisine L., Fisher G., Kovacs G., Brewer D., Clark J., Flohr P., Edwards S., on behalf of the Transatlantic Prostate Group (2010). Molecular characterisation of ERG, ETV1 and PTEN gene loci identifies patients at low and high risk of death from prostate cancer. Br. J. Cancer.

[25] Chen Y., Chi P., Rockowitz S., Iaquinta P.J., Shamu T., Shukla S., Gao D., Sirota I., Carver B.S., Wongvipat J. (2013). ETS factors reprogram the androgen receptor cistrome and prime prostate tumorigenesis in response to PTEN loss. Nat. Med..

[26] Grupp K., Kohl S., Sirma H., Simon R., Steurer S., Becker A., Adam M., Izbicki J., Sauter G., Minner S. (2013). Cysteine-rich secretory protein 3 overexpression is linked to a subset of PTEN-deleted ERG fusion-positive prostate cancers with early biochemical recurrence. Mod. Pathol..

[27] Sowalsky A.G., Ye H., Bubley G.J., Balk S.P. (2013). Clonal Progression of Prostate Cancers from Gleason Grade 3 to Grade 4. Cancer Res..

[28] Han B., Mehra R., Lonigro R.J., Wang L., Suleman K., Menon A., Palanisamy N., A Tomlins S., Chinnaiyan A.M., Shah R.B. (2009). Fluorescence in situ hybridization study shows association of PTEN deletion with ERG rearrangement during prostate cancer progression. Mod. Pathol..

[29] Morais C., Gurgel D., Teixeira A., Mattos T.A., Da Silva A.A., Tavora F. (2019). Prevalence of ERG expression and PTEN loss in a Brazilian prostate cancer cohort. Braz. J. Med. Biol. Res..

[30] Yoshimoto M., Ding K., Sweet J.M., Ludkovski O., Trottier G., Song K.S., Joshua A.M., E Fleshner N., A Squire J., Evans A.J. (2013). PTEN losses exhibit heterogeneity in multifocal prostatic adenocarcinoma and are associated with higher Gleason grade. Mod. Pathol..

[31] Bismar T.A., Yoshimoto M., Vollmer R.T., Duan Q., Firszt M., Corcos J., Squire J.A. (2010). PTEN genomic deletion is an early event associated with ERG gene rearrangements in prostate cancer. BJU Int..

[32] Bhalla R., Kunju L.P., A Tomlins S., Christopherson K., Cortez C., Carskadon S., Siddiqui J., Park K., Mosquera J.M., A Pestano G. (2013). Novel dual-color immunohistochemical methods for detecting ERG–PTEN and ERG–SPINK1 status in prostate carcinoma. Mod. Pathol..

[33] Albero-González R., Hernández-Llodrà S., Juanpere N. (2019). Immunohistochemical expression of mismatch repair proteins (MSH2, MSH6, MLH1, and PMS2) in prostate cancer: Correlation with grade groups (WHO 2016) and ERG and PTEN status. Virchows Arch..

[34] Fragkoulis C., Glykas I., Tzelves L. (2022). Clinical impact of ERG and PTEN status in prostate cancer patients underwent radical prostatectomy. Arch. Ital. Urol. Androl..

[35] Haney N.M., Faisal F.A., Lu J., Guedes L.B., Reuter V.E., Scher H.I., Eastham J.A., Marchionni L., Joshu C., Gopalan A. (2020). *PTEN* Loss with *ERG* Negative Status is Associated with Lethal Disease after Radical Prostatectomy. J. Urol..

[36] Haffner M.C., Zwart W., Roudier M.P., True L.D., Nelson W.G., Epstein J.I., De Marzo A.M., Nelson P.S., Yegnasubramanian S. (2020). Genomic and phenotypic heterogeneity in prostate cancer. Nat. Rev. Urol..

[37] Schneider T.M., Osunkoya A. (2014). ERG expression in intraductal carcinoma of the prostate: Comparison with adjacent invasive prostatic adenocarcinoma. Mod. Pathol..

[38] Sengupta P., Hospital C.C.C., Taur N., Ranjan R., Vardhan R. (2019). Study of Serum PSA, AMACR, P63 And PTEN in Prostatic Adenocarcinoma. Ann. Pathol. Lab. Med..

[39] Fu P., Bu C., Cui B., Li N., Wu J. (2021). Screening of differentially expressed genes and identification of AMACR as a prognostic marker in prostate cancer. Andrologia.

[40] Prostate Cancer: Statistics|Cancer.Net. https://www.cancer.net/cancer-types/prostate-cancer/statistics.

